# Supplementary material for: Precision Medicine Approach Based on Molecular Alterations for Patients with Relapsed or Refractory Multiple Myeloma: Results from the MM-EP1 Study
Source: Cancers (Basel). 2023 Feb 28;15(5):1508. doi: 10.3390/cancers15051508 (PMC10001403; doi:10.3390/cancers15051508)
Supplement: Supplementary file 1 [file cancers-15-01508-s001.zip › cancers-2108292-supplementary.pdf]

cancers

MDPI

Supplementary

Precision medicine approach based on molecular alteration for patients with relapsed or refractory multiple myeloma: results from MM-EP1 study

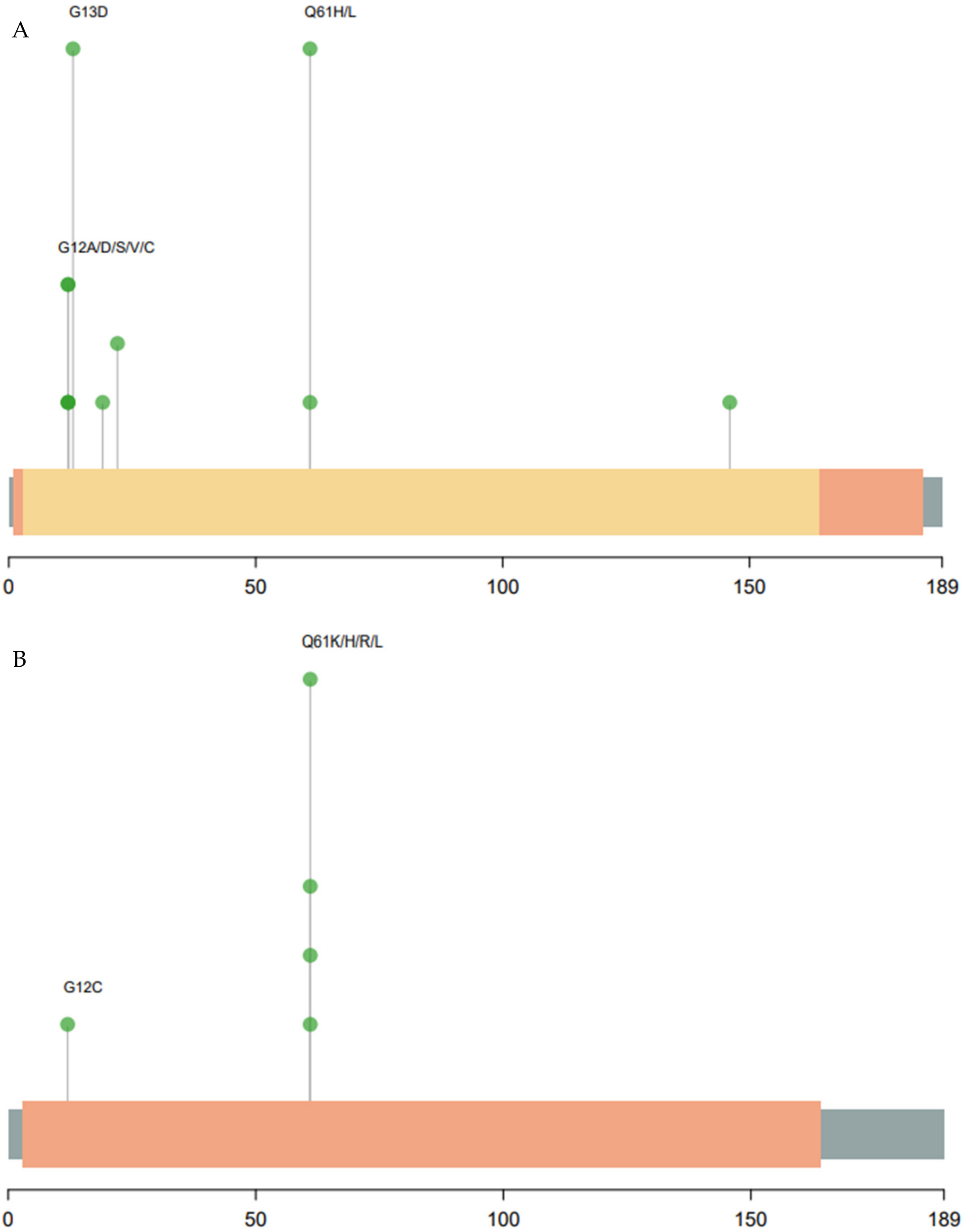

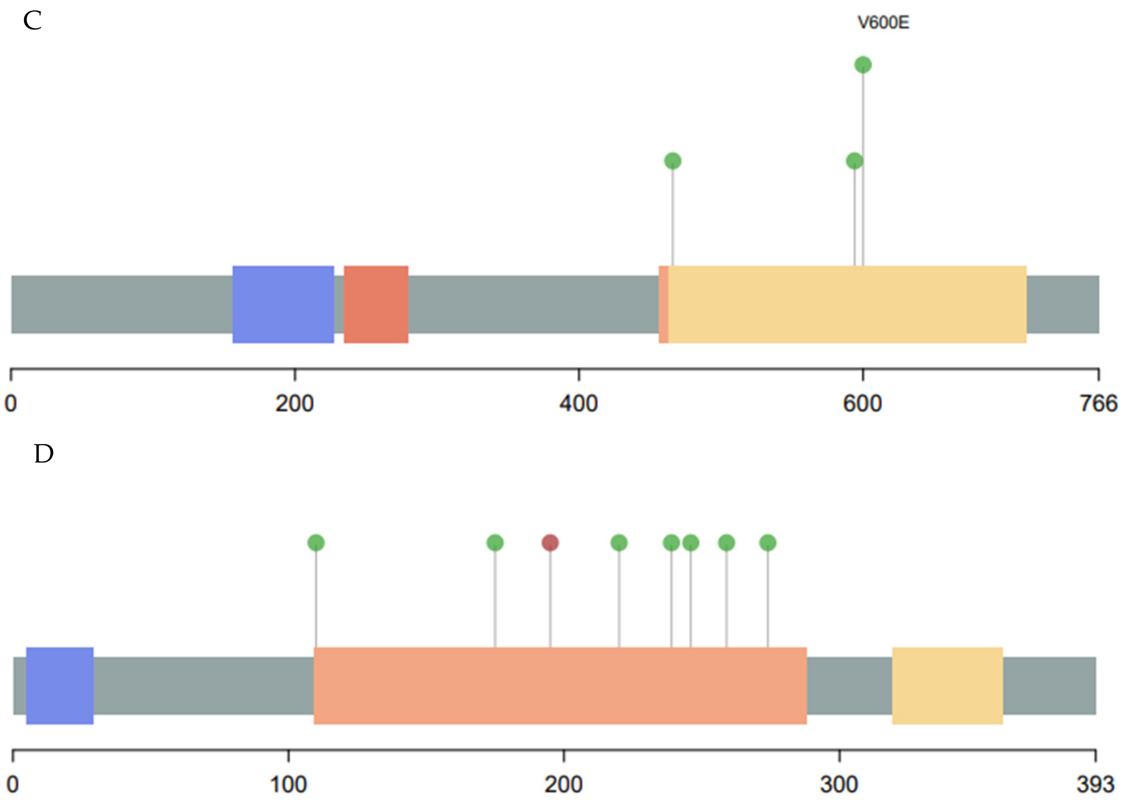

Figure S1. Lollipop diagram of the genes of interest and recurrently altered *KRAS*, *NRAS*, *BRAF* and *TP53*, in relapsed or refractory multiple myeloma patients included in the MM-EP1 study. (A). Lollipop graph – Mutations detected in *KRAS* gene (NM_033360 transcript). (B). Lollipop graph – Mutations detected in *NRAS* gene (NM_002524 transcript). (C). Lollipop graph – Mutations detected in *BRAF* gene (NM_001374258.1transcript). (D). Lollipop graph – Mutations detected in *TP53* gene (NM_000546 transcript).

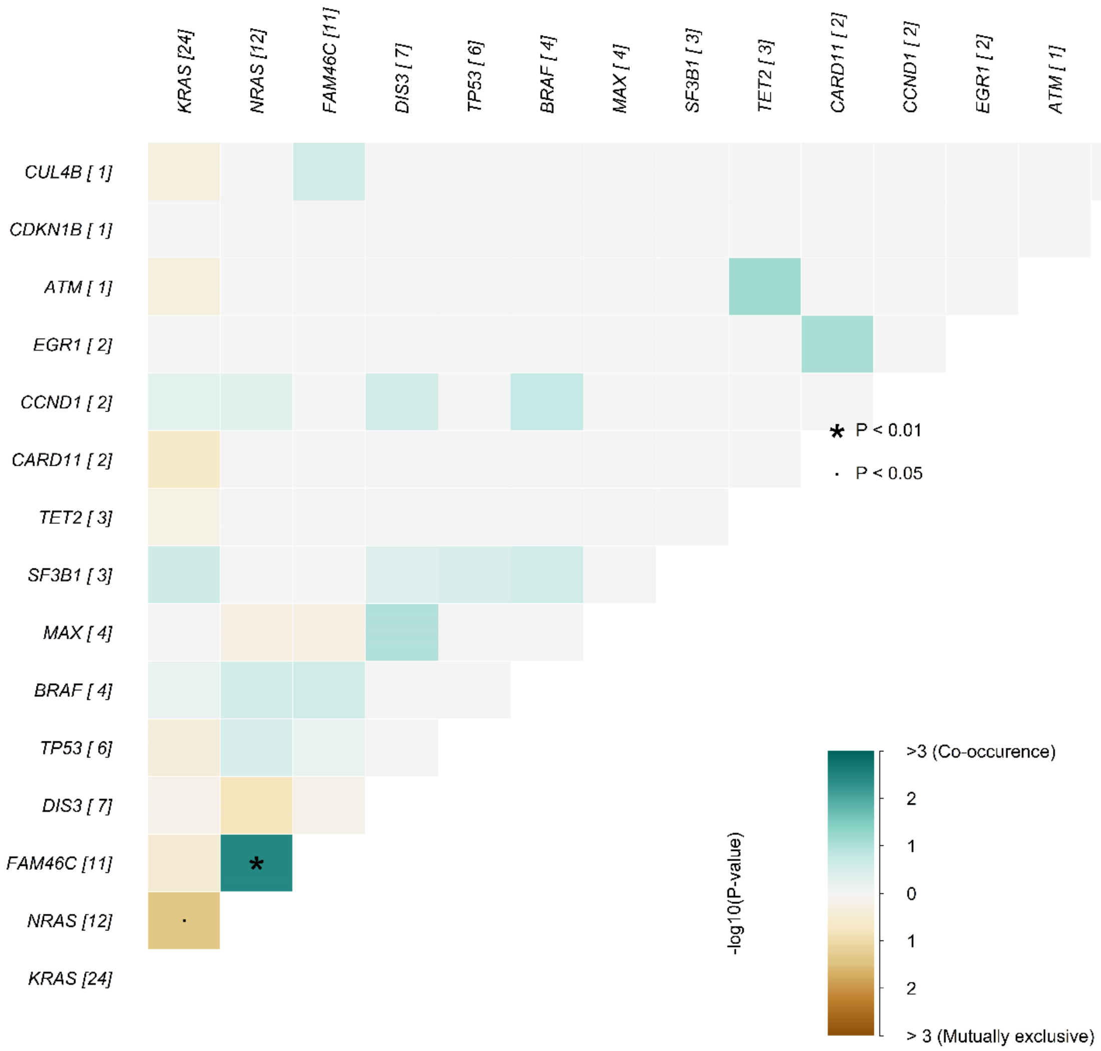

Figure S2. Correlation matrix of mutations in relapsed or refractory multiple myeloma of patients included in the MM-EP1 study. Correlation matrix analysis revealed that *NRAS* and *KRAS* mutations were mutually exclusive (p< 0.01). *FAM46C* mutations co-occur with *NRAS* (p<0.05).

Table S1. List of 29 genes included in NGS panel analysis.

ATM	EGR1	PIK3CA
BIRC3	FAM46C	PRDM1
BRAF	FGFR3	RB1
CARD11	IRF4	SF3B1
CCND1	KDM6A	SMARCB1
CDKN1B	KRAS	TET2
CRBN	LTB	TP53
CUL4A	MAX	TRAF3
CUL4B	MYC	XBP1
DIS3	NRAS	

Table S2. Somatic variants identified by NGS target sequencing in RR-MM patients included in MM-EP1.

sample no.	position	gene	HGVS_Coding	HGVS_Protein	type	AF (%)
MM01	chr12:25380275	*KRAS*	c.183A>C	p.Gln61His	MS	52
MM02	chr1:118166324	*FAM46C*	c.836del	p.Pro279ArgfsTer30	FST	46
MM02	chr1:115256530	*NRAS*	c.181C>A	p.Gln61Lys	MS	44
MM02	chr17:7578190	*TP53*	c.659A>G	p.Tyr220Cys	MS	89
MM03	chr13:73340110	*DIS3*	c.1970G>A	p.Arg657Lys	MS	20
MM03	chr1:118166340	*FAM46C*	c.850C>T	p.Gln284Ter	NS	49
MM04	chr5:137802673	*EGR1*	c.535G>T	p.Ala179Ser	MS	30
MM04	chr12:25398284	*KRAS*	c.35G>C	p.Gly12Ala	MS	42
MM04	chr13:48951053	*RB1*	c.1216-1G>A	/	splice	79
MM05	chr13:73345062	*DIS3*	c.1735A>G	p.Lys579Glu	MS	93
MM06	chrX:119694347	*CUL4B*	c.201dupT	p.Asn68Ter	FSE	44
MM06	chr1:118166255	*FAM46C*	c.765C>A	p.Phe255Leu	MS	18
MM06	chr14:103371870	*TRAF3*	c.1456C>T	p.Leu486Phe	MS	5.5
MM06	chrX:119694465	*CUL4B*	c.83G>A	p.Ser28Asn	MS	3.2
MM07	chr13:73355826	*DIS3*	c.137_145del	p.Pro46_Glu49delinsGln	IT	10
MM07	chr12:25380275	*KRAS*	c.183A>C	p.Gln61His	MS	12
MM07	chr14:65560493	*MAX*	c.104G>C	p.Arg35Pro	MS	10
MM08	chr14:65544714	*MAX*	c.211_212del	p.Ile71ProfsTer15	FST	73
MM08	chr12:25398281	*KRAS*	c.38G>A	p.Gly13Asp	MS	43
MM09	chr11:69456178	*CCND1*	c.97A>C	p.Lys33Gln	MS	9.1
MM09	chr12:25398281	*KRAS*	c.38G>A	p.Gly13Asp	MS	8.7
MM09	chr13:73355129	*DIS3*	c.241G>A	p.Glu81Lys	MS	5.4
MM09	chr13:73346883	*DIS3*	c.1334C>G	p.Ser445Ter	NS	5.6
MM11	chr4:106158403	*TET2*	c.3305del	p.Asn1102IlefsTer4	FST	29
MM11	chr4:106180851	*TET2*	c.3880_3890del	p.Tyr1294MetfsTer2	FST	28
MM11	chr11:108121531	*ATM*	c.1339C>T	p.Arg447Ter	NS	31
MM13	chr1:118166220	*FAM46C*	c.732_734del	p.Leu245del	IT	11
MM13	chr1:115256528	*NRAS*	c.183A>C	p.Gln61His	MS	5.3
MM16	chr13:73355987	*DIS3*	c.-17C>T	/	3'UTR	9.6
MM16	chr1:118165725	*FAM46C*	c.235_236del	p.Val79PhefsTer30	FST	20
MM16	chr1:115256528	*NRAS*	c.183A>C	p.Gln61His	MS	26
MM16	chr1:118166022	*FAM46C*	c.532G>A	p.Glu178Lys	MS	5

MM16	chr2:198267445	*SF3B1*	c.1912G>A	p.Ala638Thr	MS	2.5
MM16	chr6:394972	*IRF4*	c.368A>G	p.Lys123Arg	MS	40
MM16	chr7:140453155	*BRAF*	c.1780G>A	p.Asp594Asn	MS	9.4
MM16	chr12:25398284	*KRAS*	c.35G>A	p.Gly12Asp	MS	39
MM17	chr12:25380275	*KRAS*	c.183A>C	p.Gln61His	MS	2.7
MM17	chr12:25398281	*KRAS*	c.38G>A	p.Gly13Asp	MS	27
MM19	chr6:31549586	*LTB*	c.208+5G>C	/	intron	41
MM20	chr12:25378561	*KRAS*	c.437C>T	p.Ala146Val	MS	1.7
MM21	chr1:118166019	*FAM46C*	c.529T>C	p.Phe177Leu	MS	49
MM21	chr12:25398285	*KRAS*	c.34G>A	p.Gly12Ser	MS	1.9
MM22	chr2:198267481	*SF3B1*	c.1876A>T	p.Asn626Tyr	MS	0.55
MM22	chr12:25398255	*KRAS*	c.64C>A	p.Gln22Lys	MS	2
MM22	chr13:73346340	*DIS3*	c.1460A>T	p.Asp487Val	MS	26
MM22	chr17:7577505	*TP53*	c.776A>T	p.Asp259Val	MS	3.1
MM22	chr17:7578407	*TP53*	c.523C>G	p.Arg175Gly	MS	3
MM23	chr1:118165924	*FAM46C*	c.434T>C	p.Val145Ala	MS	98
MM23	chr7:140481411	*BRAF*	c.1397G>C	p.Gly466Ala	MS	4.2
MM23	chr12:25398281	*KRAS*	c.38G>A	p.Gly13Asp	MS	66
MM25	chr1:115256528	*NRAS*	c.183A>C	p.Gln61His	MS	39
MM25	chr1:115256529	*NRAS*	c.182A>G	p.Gln61Arg	MS	1.5
MM25	chr1:118166312	*FAM46C*	c.822C>A	p.Phe274Leu	MS	37
MM25	chr12:25380276	*KRAS*	c.182A>T	p.Gln61Leu	MS	4.6
MM26	chr1:115256528	*NRAS*	c.183A>T	p.Gln61His	MS	37
MM26	chr17:7577117	*TP53*	c.821T>A	p.Val274Asp	MS	6.8
MM27	chr12:25380275	*KRAS*	c.183A>C	p.Gln61His	MS	1.2
MM27	chr12:25398281	*KRAS*	c.38G>A	p.Gly13Asp	MS	1.3
MM27	chr12:25398284	*KRAS*	c.35G>A	p.Gly12Asp	MS	41
MM31	chr1:115258748	*NRAS*	c.34G>T	p.Gly12Cys	MS	3.8
MM31	chr1:118166385	*FAM46C*	c.895G>A	p.Glu299Lys	MS	50
MM31	chr17:7577565	*TP53*	c.716A>G	p.Asn239Ser	MS	99
MM32	chr13:73351626	*DIS3*	c.586G>A	p.Glu196Lys	MS	69
MM32	chr14:65544690	*MAX*	c.236A>G	p.His79Arg	MS	2.8
MM36	chr12:25398284	*KRAS*	c.35G>A	p.Gly12Asp	MS	47
MM36	chr2:198267361	*SF3B1*	c.1996A>G	p.Lys666Glu	MS	0.4
MM37	chr1:115256529	*NRAS*	c.182A>T	p.Gln61Leu	MS	2
MM37	chr7:140453136	*BRAF*	c.1799T>A	p.Val600Glu	MS	7
MM37	chr11:69456132	*CCND1*	c.51_52delCCinsTT	p.Pro18Ser	MS	22
MM37	chr11:69456255	*CCND1*	c.174G>C	p.Lys58Asn	MS	17
MM37	chr12:25398281	*KRAS*	c.38G>A	p.Gly13Asp	MS	10
MM38	chr5:137802862	*EGR1*	c.724G>A	p.Ala242Thr	MS	4.2
MM38	chr7:2977569	*CARD11*	c.1115T>C	p.Leu372Pro	MS	0.7
MM40	chr1:118165630	*FAM46C*	c.140_142del	p.Thr47_Leu48delinsMet	IT	20
MM40	chr1:115256529	*NRAS*	c.182A>G	p.Gln61Arg	MS	48
MM42	chr12:12870975	*CDKN1B*	c.204dupA	p.Pro69ThrfsTer56	FSE	30
MM42	chr12:25398262	*KRAS*	c.57G>C	p.Leu19Phe	MS	3.5
MM43	chr1:118166223	*FAM46C*	c.733del	p.Leu245SerfsTer22	FST	4.4
MM43	chr4:106196891	*TET2*	c.5224_5227del	p.Pro1742IlefsTer2	FST	1.5
MM43	chr1:115256528	*NRAS*	c.183A>C	p.Gln61His	MS	2.5
MM44	chr12:25380275	*KRAS*	c.183A>C	p.Gln61His	MS	43

MM45	chr13:73352350	*DIS3*	c.555A>G	p.Ile185Met	MS	2.7
MM47	chr12:25380275	*KRAS*	c.183A>C	p.Gln61His	MS	38
MM48	chr1:115256528	*NRAS*	c.183A>C	p.Gln61His	MS	50
MM49	chr7:140453136	*BRAF*	c.1799T>A	p.Val600Glu	MS	15
MM51	chr12:25398284	*KRAS*	c.35G>T	p.Gly12Val	MS	12
MM52	chr12:25398281	*KRAS*	c.38G>A	p.Gly13Asp	MS	35
MM53	chr12:25380275	*KRAS*	c.183A>C	p.Gln61His	MS	33
MM55	chr1:115256529	*NRAS*	c.182A>G	p.Gln61Arg	MS	47
MM56	chr14:65560491	*MAX*	c.106A>T	p.Arg36Trp	MS	71
MM57	chr4:106180817	*TET2*	c.3845G>T	p.Gly1282Val	MS	1.7
MM57	chr12:25398284	*KRAS*	c.35G>T	p.Gly12Val	MS	47
MM57	chr4:106183003	*TET2*	c.4042C>T	p.Gln1348Ter	NS	1.2
MM59	chr17:7578244	*TP53*	c.582_605del	p.Ile195_Arg202del	IT	30
MM59	chr17:7579358	*TP53*	c.329G>C	p.Arg110Pro	MS	9
MM61	chr17:7577543	*TP53*	c.738G>T	p.Met246Ile	MS	3.6
MM61	chr12:25398284	*KRAS*	c.35G>T	p.Gly12Val	MS	47
MM61	chr12:25398255	*KRAS*	c.64C>A	p.Gln22Lys	MS	50
MM62	chr12:25398285	*KRAS*	c.34G>T	p.Gly12Cys	MS	24
MM63	chr22:24134063	*SMARCB1*	c.214A>T	p.Thr72Asn	MS	3.8
MM63	chr7:2979516	*CARD11*	c.731A>T	p.Lys244Met	MS	4.5
MM66	chr1:115256529	*NRAS*	c.182A>T	p.Gln61Leu	MS	42

chr: chromosome, HGVS: Human Genome Variation Society, MS: missense variant, NS: nonsense variant, IT: inframe truncation: FSE: frameshift elongation, FST: frameshift truncation, AF: allelic frequency. Indicated cDNA change referred to following transcripts: NM_000051.3 (*ATM*), NM_001374258.1 (*BRAF*), NM_001324281.1 (*CARD11*), NM_053056.3 (*CCND1*), NM_004064.5 (*CDKN1B*), NM_003588.3 (*CUL4B*), NM_014953.5 (*DIS3/RRP44*), NM_001964.3 (*EGR1*), NM_017709.4 (*FAM46C/TENT5C*), NM_002460.4 (*IRF4/MUM1*), NM_033360.4 (*KRAS*), NM_002341.2 (*LTB*), NM_002382.5 (*MAX*), NM_002524.5 (*NRAS*), NM_000321.2 (*RB1*), NM_012433.4 (*SF3B1*), NM_003073.3 (*SMARCB1/INI1*), NM_001127208.3 (*TET2*), NM_000546.5 (*TP53*), NM_003300.4 (*TRAF3*).
